# Donor-dependent heterogeneity and matrix-remodeling transcriptional programs in *COL2A1*-variant urine-derived cell cultures with IGF1-associated cell-state shifts

**DOI:** 10.3389/fcell.2026.1920598

**Published:** 2026-09-01

**Authors:** Alexander Schulz, Michael Schubert, Emily M. Brockmann, Arif B. Ekici, Steffen Uebe, Christian T. Thiel

**Affiliations:** Institute of Human Genetics, Centre for Personalised Medicine and Research (CESAR), Friedrich-Alexander University Erlangen-Nürnberg, Erlangen, Germany

**Keywords:** chondrogenesis, *COL2A1*, endochondral ossification, extracellular matrix, growth plate, IGF1, single-cell RNA sequencing, urine-derived stem cells

## Abstract

*COL2A1*-associated skeletal dysplasias affect cartilage extracellular matrix and endochondral growth, but patient growth-plate tissue is largely inaccessible. We generated chondrogenically induced urine-derived cells (chUDCs) from three individuals with heterozygous pathogenic *COL2A1* variants and three healthy controls. Classical differentiation was assessed by Alcian blue and Alizarin red staining, bulk RNA sequencing compared patient-derived and control chUDCs, and single-cell RNA sequencing was performed in two patient-derived lines. *COL2A1*-mutant chUDCs retained overt chondrogenic and osteogenic staining capacity without a clear patient-control separation. In contrast, bulk RNA sequencing identified a small set of consistently differential extracellular-matrix remodeling genes enriched for matrix and ossification-related programs; *DDR2* and *ADAMTS5* remained significant in leave-one-donor-out, age-adjusted and cell-composition-adjusted models. Single-cell profiling resolved multiple chUDC states, including chondrogenic, fibroblastic extracellular-matrix, IGF1R-high, and osteogenic-associated states. IGF1 co-treatment was associated with reduced osteogenic-associated module scores and increased fibroblastic/anabolic extracellular-matrix programs. Because no IGF1-treated control-donor line was profiled, IGF1-associated changes are reported as exploratory and are not claimed to be *COL2A1*-specific. These findings support chUDCs as a non-invasive system for exploring selected transcriptional programs relevant to cartilage biology and growth-factor responsiveness in *COL2A1*-associated growth disorders.

## 1 Introduction

Heterozygous variants in *COL2A1*, encoding the α1 chain of type II collagen, cause type II collagenopathies ranging from Stickler syndrome to disproportionate short stature and skeletal dysplasia (Gregersen and Savarirayan, 2026; Zhang et al., 2020). Because type II collagen is a major cartilage extracellular-matrix component, these disorders affect cartilage integrity, endochondral growth, and linear growth (Eyre, 2002; Mackie et al., 2008).

A central obstacle is that the disease-relevant human growth plate is not available for routine molecular analysis. Animal models, induced pluripotent stem cell systems, and cartilage organoids provide important platforms (Lamande et al., 2023), but can be time-consuming, technically demanding, or difficult to apply systematically to small cohorts with rare private variants.

Urine-derived cells (UDCs), which contain a multipotent stem/progenitor population, provide an accessible non-invasive cell source that can be expanded *in vitro* and directed toward mesenchymal differentiation programs (Bharadwaj et al., 2013; Sun et al., 2021). Here, we refer to chondrogenically induced urine-derived cells as chUDCs.

The growth hormone/insulin-like growth factor 1 axis is a central endocrine regulator of postnatal growth and cartilage biology (Racine and Serrat, 2020; Dixit et al., 2021). IGF1 promotes chondrocyte proliferation, matrix production, and anabolic cartilage programs in experimental systems, although clinical growth-hormone responses across skeletal dysplasias are variable, and evidence for rarer disorders remains limited (Kochar and Chugh, 2020). For *COL2A1*-associated growth disorders, it remains unclear whether patient-derived cells contain IGF1-responsive transcriptional states outside the native growth plate.

To sample a clinically meaningful spectrum of pathogenic *COL2A1* variants rather than a single genotype, we studied urine-derived cells from three affected individuals carrying distinct variant classes alongside three unaffected controls. Here, we combined conventional differentiation assays, bulk RNA sequencing, and single-cell RNA sequencing to ask whether *COL2A1*-variant chUDCs show extracellular-matrix or ossification-associated transcriptional programs not apparent from endpoint staining, and whether paired single-cell profiling in two patient-derived lines identifies IGF1-responsive transcriptional states.

## 2 Methods

### 2.1 Participants and genetic variants

Urine samples were obtained from three individuals with clinically classified pathogenic heterozygous *COL2A1* variants and from three healthy control donors after written informed consent. Patient P1 carried *COL2A1* (NM_001844.5) c.2381del, p(Pro794LeufsTer), dominant with unknown inheritance, and had Stickler syndrome; P2 carried *COL2A1* (NM_001844.5) c.908G>A, p(Gly303Asp), dominant maternal inheritance, and had disproportionate short stature; and P3 carried *COL2A1* (NM_001844.5) c.2032G>A, p(Gly678Arg), *de novo*, and had mild disproportionate short stature. Variants were identified by clinical exome sequencing in two individuals and genome sequencing in one individual and classified as pathogenic according to ACMG criteria (Richards et al., 2015). Controls C1–C3 were healthy donors and were not age-matched to patients (controls 30–34 years; patients 5–28 years). Donor sex and age are summarized in Supplementary Table S1. C1–C3 constituted the primary control cohort used for bulk RNA sequencing and conventional differentiation assays. An additional healthy control line, C4, was used only in the qPCR marker-expression and type II collagen immunofluorescence experiments. Because donor age and disease status were strongly collinear in this cohort (point-biserial r = −0.77), age was not included as a covariate in the primary differential-expression model; age-adjusted sensitivity analyses are reported separately (Supplementary Table S9).

### 2.2 Isolation, expansion, and chondrogenic induction of urine-derived cells

UDCs were isolated from freshly collected urine using a modified urine-cell culture protocol (Bharadwaj et al., 2013; Bharadwaj et al., 2011). Samples were processed as soon as possible, centrifuged, washed, resuspended in expansion medium, seeded on 0.2% gelatin-coated culture vessels, and expanded in α-MEM Eagle medium with 20% fetal bovine serum, 5 ng/mL basic fibroblast growth factor, and 1% penicillin/streptomycin at 37 °C and 5% CO_2_. Cells were passaged with trypsin, expanded before or around passage 7, cryopreserved, and thawed for differentiation and sequencing. The transcript expression of mesenchymal- and hematopoietic-associated markers in undifferentiated UDCs was characterized by quantitative PCR. The control lines used for marker-expression analysis and type II collagen immunofluorescence (C1, C2, and C4) were derived from a single expansion batch and received a transient treatment with the Rho-associated kinase inhibitor Y-27632 (10 µM) for 48–72 h during passaging to support cell survival and attachment; the patient lines were not exposed to the inhibitor. Y-27632 was applied only transiently during expansion and initial harvest and was never present in the chondrogenic differentiation medium. The cell batches used for bulk and single-cell RNA sequencing were expanded before this practice was introduced and were therefore not exposed to the inhibitor at any point.

For chondrogenic induction, UDCs were cultured as two-dimensional monolayers on 0.2% gelatin using the StemPro Chondrogenesis Differentiation Kit supplemented with 1% penicillin/streptomycin. Medium was changed three times per week. For bulk RNA sequencing, 200,000 cells were seeded per 24-well; for staining assays, 50,000 cells were seeded per 96-well. For single-cell RNA sequencing, near-confluent T25 cultures were used. In this study, chondrogenically induced UDCs are referred to as chUDCs after at least 2 weeks of induction. Bulk RNA sequencing and the corresponding conventional staining assays were performed after 3 weeks of chondrogenic induction, whereas scRNA-seq samples were harvested after 2 weeks.

### 2.3 Mesenchymal and hematopoietic marker-expression analysis by quantitative PCR

Total RNA from undifferentiated UDCs was extracted using a column-based kit with on-column genomic DNA removal and reverse-transcribed with LunaScript RT SuperMix (New England Biolabs). Relative expression of mesenchymal and hematopoietic marker transcripts–*NT5E* (CD73), *THY1* (CD90), *ENG* (CD105), *CD44*, *PTPRC* (CD45), and *CD34* – was quantified with predesigned TaqMan assays (Applied Biosystems) on a QuantStudio 12K Flex system, using *ACTB*, *B2M*, *RPLP0*, and *PGK1* as reference genes. Raw Ct values (four technical replicates per sample) were processed in R (v4.4.3); replicates with Ct > 35 were treated as non-detected, and per-sample Ct was averaged across the remaining replicates and normalized to the mean of the four reference genes to obtain ΔCt. Relative quantities (RQ, 2^−ΔΔCt^) were referenced within each sample to *CD44* (RQ = 1). Marker expression was compared between control (n = 3) and patient (n = 3) lines per gene using unpaired two-sided Welch’s t-tests on ΔCt values; genes detected in fewer than two lines per group were reported descriptively without testing.

### 2.4 Osteogenic differentiation and IGF1 treatment

Osteogenic differentiation was initiated from expanded UDCs seeded on 0.2% gelatin at 50,000 cells per 96-well and cultured for 3 weeks using the StemPro Osteogenesis Differentiation Kit. Medium was changed three times per week.

For IGF1 co-treatment experiments, recombinant human IGF1 (PeproTech; Cat. 100-11-500UG) was added to the chondrogenic differentiation medium at 150 ng/mL for the full duration of differentiation. This concentration was selected because 150 ng/mL gave maximal induction of chondrogenic markers (*SOX9*, *COL2A1*) in mesenchymal stromal cells when compared directly against 75 and 300 ng/mL (Widowati et al., 2018). IGF1 co-treatment was applied to the P1 and P2 scRNA-seq arm and to matched staining cultures, but not to the bulk RNA-seq patient-control comparison. Control cultures received chondrogenic differentiation medium without IGF1.

### 2.5 Histological staining and imaging

Alcian blue staining was performed after fixation with 4% paraformaldehyde for 30 min, PBS washing, and 1 h staining with 1% Alcian Blue 8GX in 3% acetic acid to visualize proteoglycan-rich glycosaminoglycan deposition, following an adapted staining protocol (Prosser et al., 2019). Alizarin red staining was performed after the same fixation, PBS washing, and 1 h staining with Alizarin Red Solution to visualize calcium deposition as an osteogenic maturation-associated readout, following an adapted staining protocol (Gregory et al., 2004). Images were acquired after three PBS washes using a Vert. A1 microscope.

For quantification, Alcian blue-stained, PBS-washed cultures were incubated overnight with 4 M guanidine hydrochloride to solubilize bound dye, and absorbance was measured in the same plate at 610 nm. For Alizarin red quantification, wells were washed with distilled water, incubated with 10% acetic acid for 30 min at room temperature, and the extract was transferred to a new plate, neutralized with NaOH, and measured at 405 nm. Per-donor absorbance values were averaged across replicate wells and compared between patient-derived and control cultures using unpaired two-sided Welch’s t-tests.

### 2.6 Type II collagen immunofluorescence

Type II collagen protein was assessed by immunofluorescence in chondrogenically induced control (C1, C2, C4) and patient-derived cultures after approximately 10 days of induction on 0.2% gelatin. Cultures were fixed for 10 min in ice-cold EGTA-saturated methanol at −20 °C, washed, permeabilized with 0.01% Tween-20 in PBS, and blocked for 1 h at room temperature. Cells were incubated overnight at 4 °C with a rabbit polyclonal anti-type II collagen antibody (Proteintech, 28459-1-AP; dilution 1:200), followed by an Alexa Fluor 488-conjugated anti-rabbit secondary antibody and DAPI counterstain. Images were acquired on a Zeiss Axio Imager 2 with Apotome under constant exposure settings (Alexa Fluor 488/GFP channel, 150 ms; DAPI, 30 ms).

### 2.7 Bulk RNA sequencing and pathway analysis

Bulk RNA sequencing was performed on chUDCs after 3 weeks of chondrogenic induction from all three patient-derived and all three control lines, with one biological sample per donor. RNA was extracted using the laboratory’s standard column-based RNA-seq workflow with genomic DNA removal, quality-controlled, converted into stranded mRNA libraries, and sequenced as paired-end reads on an Illumina NovaSeq X Plus platform. Reads were demultiplexed and filtered; filtered reads were processed with bwa mem and samtools, converted back to FASTQ using GATK SamToFastq, aligned to hg38 with STAR, and summarized as gene-level counts with featureCounts (Li and Durbin, 2009; Li et al., 2009; Van der Auwera et al., 2013; Dobin et al., 2013; Liao et al., 2014); downstream analysis code is available in the public repository.

Differential expression analysis was performed in R using DESeq2 (Love et al., 2014). Genes with at least 10 counts in at least three samples were retained, and the model compared patient-derived versus control chUDCs. Fold changes were shrinkage-estimated using ashr, and Benjamini–Hochberg adjusted p values <0.05 were considered statistically significant (Stephens, 2017; Benjamini and Hochberg, 1995). Gene-set enrichment analysis was performed using clusterProfiler with Gene Ontology Biological Process terms on a signed ranking metric based on log_2_ fold-change direction and nominal p value (Wu et al., 2021; Gene Ontology Consortium, 2021). Full differential-expression and gene-set enrichment result tables are provided as Supplementary Tables S2, S3.

### 2.8 Sensitivity analyses of the bulk signature

Three sensitivity analyses were applied to the patient-versus-control model, each retaining the primary model’s gene universe so that runs remained comparable. First, six leave-one-donor-out models were fitted, each omitting one donor. Second, the model was refitted with age as a covariate, both continuous and as a pediatric/adult factor. Third, a signature matrix was built from the annotated single-cell states (untreated cells, ≥20 cells per state), cell-state proportions were estimated per bulk sample by non-negative least squares on the linear scale (Newman et al., 2015), and their first principal component was included as a covariate; with six samples only one composition covariate was admissible. Results are given in Supplementary Tables S8, S9, S11.

### 2.9 Single-cell RNA sequencing and cell-state analysis

Single-cell RNA sequencing was performed on P1 and P2 chUDCs under paired control and IGF1-treated conditions after 2 weeks of chondrogenic induction. Libraries were generated using the 10x Genomics GEM-X 3′v4 OCM 4-plex workflow. Libraries were sequenced on the Illumina NovaSeq X Plus platform. FASTQ generation and demultiplexing followed the 10x Genomics workflow, and the resulting FASTQ files were processed using Cell Ranger Multi v10.0.0 with a GRCh38 reference genome (Zheng et al., 2017; Cell Ranger, 2026).

Downstream analysis was performed in Seurat v5 (Hao et al., 2024). Raw matrices were imported using Read10X_h5, per-cell mitochondrial and ribosomal percentages were calculated, and cells were filtered using sample-specific thresholds for detected genes, UMI counts, and mitochondrial content (Supplementary Table S4). Doublets were identified with scDblFinder and removed before integrated singlet analysis (Germain et al., 2021).

Filtered cells were log-normalized, the 2,000 most variable features were selected with the vst method, and principal component analysis was performed using 50 PCs. For joint visualization and clustering, donor-aware integration was performed with Harmony using donor as the integration variable while preserving IGF1 treatment as the biological condition of interest (Korsunsky et al., 2019). UMAP embedding and Louvain clustering were performed using dimensions 1–20 at resolution 0.5 (McInnes et al., 2018; Blondel et al., 2008). Cluster markers were identified using Wilcoxon rank-sum testing with FindAllMarkers, retaining positive markers expressed in at least 25% of cells with a log_2_ fold-change threshold of 0.25. Cell states were annotated by combining cluster markers with curated module scores for chondrogenic, cartilage extracellular-matrix, IGF receptor, IGFBP, MAPK/immediate-early, heat-shock, hypertrophic, osteogenic, stromal, fibroblastic extracellular-matrix, cell-cycle, and inflammatory programs. Module scores were calculated using AddModuleScore (Tirosh et al., 2016). Complete marker tables, module gene sets, and literature support for the annotation scheme are provided in Supplementary Table S5. For the single-cell IGF1-response differential expression analysis, raw counts were aggregated into sample-level pseudobulk profiles for P1, P1-IGF1, P2, and P2-IGF1, and differential expression was assessed with DESeq2 using a paired design accounting for donor (∼ donor + condition). Genes with at least 10 total counts and expression in at least two samples were retained, and Benjamini–Hochberg-adjusted p values <0.05 with absolute log_2_ fold changes ≥1 were used for visualization. Given the inclusion of only two donor-derived lines, these results were interpreted exploratorily. To assess whether IGF1-associated changes were cell-intrinsic rather than shifts in cell-state proportions, counts were aggregated per donor, cell state and treatment, and differential expression was tested within each cell state using edgeR quasi-likelihood F-tests with a donor-paired design (Robinson et al., 2010). Only states with at least 20 cells in all four donor-by-treatment groups were tested. Results are provided as Supplementary Table S10.

### 2.10 Statistical analysis and reproducibility

For donor-level bulk RNA-seq comparisons, the biological unit was the donor-derived cell line. Single-cell analyses were interpreted as exploratory state-resolved analyses because only two patient-derived lines were profiled without an IGF1-treated control-donor arm. No formal power calculation was performed because the study was designed as an exploratory proof-of-concept analysis of rare patient-derived lines. Analysis scripts are available at https://github.com/alexschulzcell/COL2A1-paper.

## 3 Results

### 3.1 *COL2A1*-mutant chUDCs show comparable chondrogenic and osteogenic staining across patient-derived and control cultures

**FIGURE 1:**
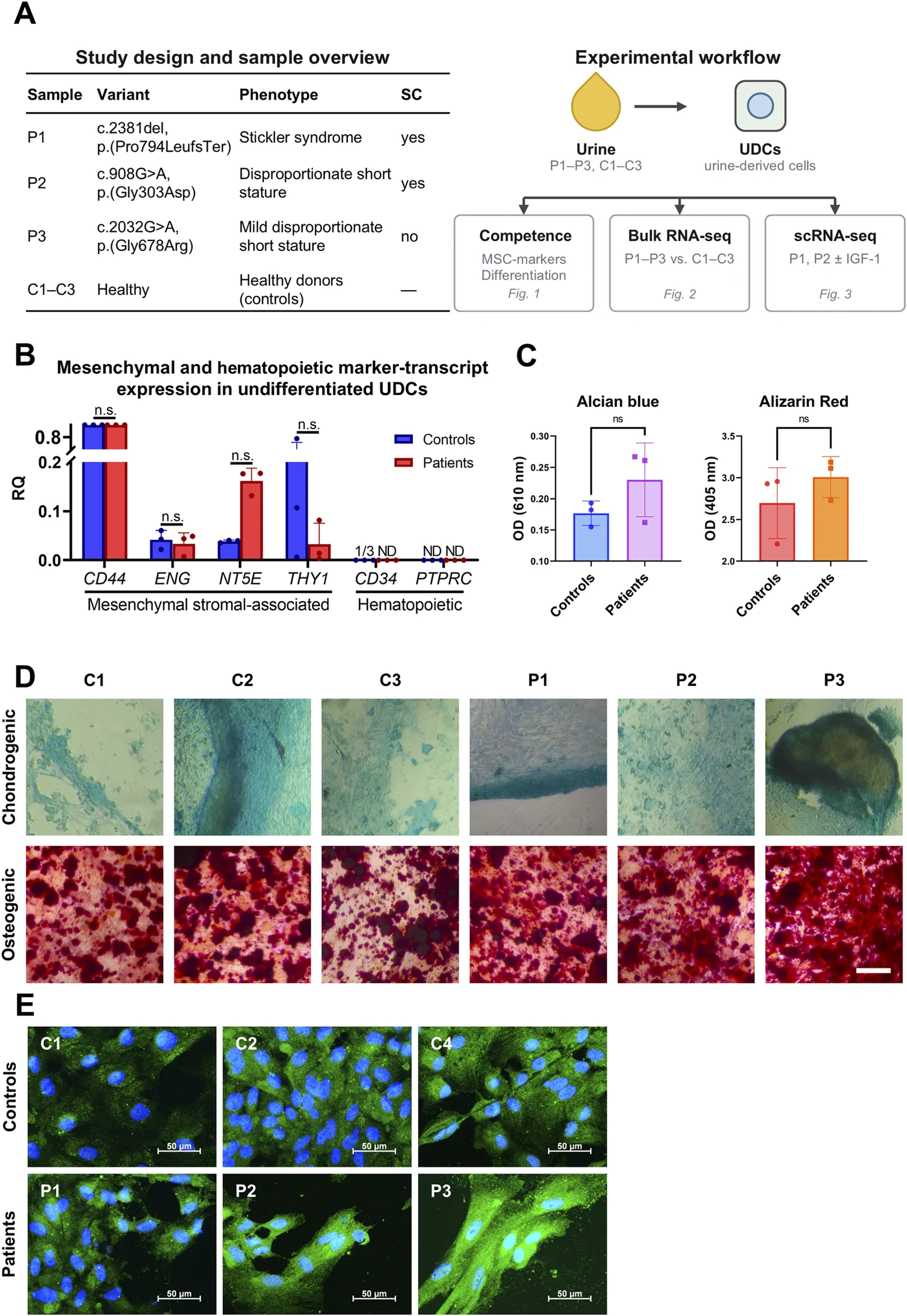
Patient-derived *COL2A1*-mutant chUDC model and conventional differentiation phenotype. **(A)** Study design and experimental workflow. Three individuals carrying pathogenic heterozygous *COL2A1* variants (P1, c.2381del, p.(Pro794LeufsTer); P2, c.908G>A, p.(Gly303Asp); P3, c.2032G>A, p.(Gly678Arg)) and three healthy donors (C1–C3) were studied; clinical short-stature (SC) status is indicated. Urine-derived cells (UDCs) were isolated from urine and analyzed along three axes: conventional chondrogenic and osteogenic differentiation, bulk RNA sequencing (P1–P3 vs. C1–C3), and single-cell RNA sequencing (P1 and P2, ± IGF1). **(B)** Mesenchymal and hematopoietic marker expression in undifferentiated UDCs by quantitative PCR: the mesenchymal stromal markers *NT5E* (CD73), *THY1* (CD90), *ENG* (CD105) and *CD44* were expressed, whereas the hematopoietic markers *PTPRC* (CD45) and *CD34* were low or undetectable. Bars show the mean relative quantity (RQ, 2^−ΔΔCt^) anchored to *CD44* (RQ = 1), with error bars indicating the SD; n/N above each bar gives the number of samples with detectable signal (Ct ≤ 35) out of the total per group, and ND denotes markers not detected in any sample. Groups were compared per gene by unpaired two-sided Welch’s t-tests on ΔCt (n = 3 controls, n = 3 patients). n. s., not significant. **(C)** Quantification of Alcian blue (610 nm) and Alizarin red (405 nm) absorbance in control (n = 3) and patient (n = 3) cultures (bars, mean ± SD); neither staining differed significantly between groups (unpaired Welch’s t-test, not significant). **(D)** Representative chondrogenic (Alcian blue) and osteogenic (Alizarin red) staining of control (C1–C3) and patient-derived (P1–P3) chUDC cultures; no qualitative patient–control difference was apparent. Scale bar, 200 µm. **(E)** Type II collagen immunofluorescence of chondrogenically induced control (C1, C2 and C4) and patient cultures with DAPI counterstain; no overt patient–control difference in *COL2A1*-encoded type II collagen signal or extracellular matrix deposition was observed. Scale bar, 50 µm.

We established chUDCs from three individuals with heterozygous *COL2A1* variants and three healthy controls. The workflow combined urine-derived UDC generation, chondrogenic and osteogenic differentiation assays, bulk RNA sequencing in all six lines, and single-cell RNA sequencing in P1 and P2 under control and IGF1-treated conditions (Figure 1A).

Undifferentiated UDCs expressed transcripts encoding the mesenchymal stromal-associated markers *NT5E* (CD73), *THY1* (CD90), *ENG* (CD105), and *CD44* by quantitative PCR, whereas the hematopoietic marker *PTPRC* (CD45) and *CD34* were low or absent, among the markers tested, no significant differences in expression were detected between patient-derived and control lines (unpaired Welch’s t-test on ΔCt, not significant; Figure 1B).

Alcian blue and Alizarin red staining showed chondrogenic matrix and mineral deposition across patient-derived and control cultures (Figure 1D). Quantification of Alcian blue (610 nm) and Alizarin red (405 nm) absorbance confirmed no significant difference between groups (unpaired Welch’s t-test, not significant; Figure 1C). Immunofluorescence for type II collagen likewise showed no overt patient-control difference in COL2A1 protein signal or deposition in chondrogenically induced cultures (Figure 1E).

### 3.2 Bulk RNA sequencing shows transcriptional differences enriched for extracellular-matrix and ossification-related gene sets

**FIGURE 2:**
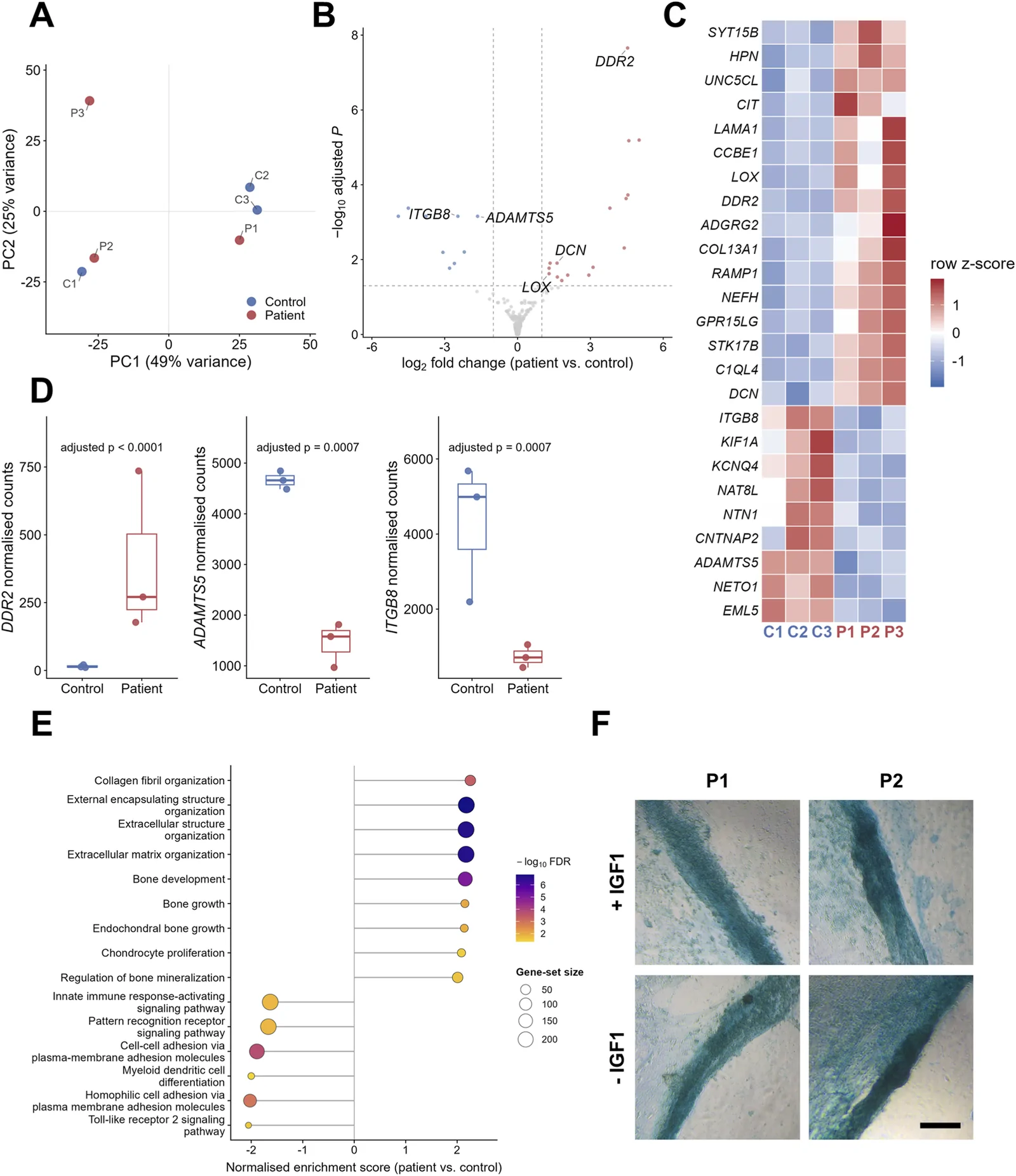
Bulk RNA sequencing shows transcriptional differences enriched for extracellular-matrix and ossification-related gene sets in *COL2A1*-variant chUDCs. **(A)** Principal component analysis of bulk transcriptomes; samples do not separate along a clear patient-control axis on PC1 (49% of variance); C1 and P2 localize together, C2/C3/P1 cluster separately, and P3 separates along PC2 (25% of variance). **(B)** Volcano plot of differential expression (patient vs. control); selected genes are labeled, including upregulated *DCN* and *DDR2* and down-regulated *ADAMTS5* and *ITGB8*. **(C)** Heatmap of significant differentially expressed genes (row-scaled z-scores) across control (C1–C3) and patient (P1–P3) samples. **(D)** Normalized count distributions in control versus patient cultures for the most robust signature genes: *DDR2* (padj < 0.0001) and *ADAMTS5* (padj = 0.0007), which remain significant in every sensitivity analysis, and *ITGB8* (padj = 0.0007), which is significant in five of six leave-one-donor-out models and after adjustment for cell-state composition. **(E)** Gene-set enrichment (normalized enrichment score, patient vs. control); positively enriched terms include collagen fibril organization, extracellular-matrix organization, endochondral bone growth and bone mineralization, whereas cell–cell adhesion and innate immune/pattern-recognition programs are negatively enriched; point colour encodes the false-discovery-rate (FDR)-adjusted p-value and point size indicates gene-set size. **(F)** Representative Alcian blue staining of P1 and P2 chUDCs cultured with or without IGF1, qualitative staining comparison for the IGF1 co-treatment condition. Scale bar, 200 µm.

Bulk RNA-seq after chondrogenic induction showed donor-dependent heterogeneity rather than a simple patient-control separation in principal component analysis (Figure 2A), warranting cautious gene- and pathway-level interpretation. PC1 and PC2 explained 49.4% and 24.9% of the variance. Sample-to-sample correlations were uniformly high (Spearman r = 0.91–0.96), and per-sample Cook’s distances gave no indication that any donor, including P3, was a technical outlier (Supplementary Table S8).

Differential expression analysis identified a limited but coherent gene signature rather than broad transcriptional dysregulation. Patient-derived chUDCs showed increased expression of matrix- and cell-matrix-associated transcripts including *DDR2*, *DCN*, *LOX*, *LAMA1*, and *COL13A1*, whereas *ADAMTS5* and *ITGB8* were reduced (Figures 2B,C). Several of these genes have established roles in cartilage-matrix signaling, aggrecan turnover, and chondrocyte–matrix interactions, including *DDR2*, *DCN*, *ADAMTS5*, and integrin-mediated signaling (Xu et al., 2005; Li et al., 2020; Loeser, 2014; Stanton et al., 2005). Normalized count distributions for *DDR2*, *ADAMTS5* and *ITGB8* are shown in Figure 2D. Each of the three patient-derived lines individually deviated from the control mean in the expected direction for all seven genes, and patient and control ranges did not overlap for any of them (Supplementary Table S8), indicating that the signature is not an average across divergent donors. The complete DESeq2 output is provided as Supplementary Table S2.

Gene-set enrichment analysis supported these gene-level findings. Patient-derived chUDCs showed positive enrichment for extracellular-matrix organization, collagen fibril organization, bone development, endochondral bone growth, chondrocyte proliferation, and regulation of bone mineralization (Figure 2E). The complete gene-set enrichment output is provided as Supplementary Table S3.

Because the cohort is small and heterogeneous, we tested the stability of this signature. Across six leave-one-donor-out models, none of the 25 genes significant in the full model changed direction, and genome-wide log_2_ fold changes remained highly correlated with the full model (Pearson r = 0.77–0.91). *DDR2* and *ADAMTS5* retained significance in all six models and *ITGB8* in five, whereas *DCN*, *COL13A1*, *LAMA1* and *LOX* retained their direction and effect size but lost significance in individual models, consistent with reduced power at n = 2 per group rather than an unstable effect. *DDR2* and *ADAMTS5* also remained significant after adjustment for age and for estimated cell-state composition, and no estimated cell-state proportion differed significantly between groups (Supplementary Tables S8, S9, S11; Supplementary Figure S4). We therefore regard *DDR2* and *ADAMTS5*, supported by *ITGB8*, as the robust core of the signature.

Alcian blue staining of P1 and P2 chUDCs with and without IGF1 co-treatment is shown in Figure 2F. Because bulk RNA sequencing averages signal across the population, we next used single-cell RNA sequencing to resolve the cellular-state architecture underlying this signature and its IGF1 responsiveness.

### 3.3 Single-cell RNA sequencing shows IGF1-associated changes in osteogenic and extracellular-matrix-associated chUDC states

Paired single-cell profiling was performed in P1 and P2 chUDCs under control and IGF1-treated conditions to test whether the bulk phenotype reflected discrete transcriptional states. Quality-control filtering retained most cells and removed low-quality cells and predicted doublets, while integration diagnostics supported improved donor mixing after correction (Supplementary Figures S1A-D, Supplementary Figures S2A-F; Supplementary Table S4).

**FIGURE 3:**
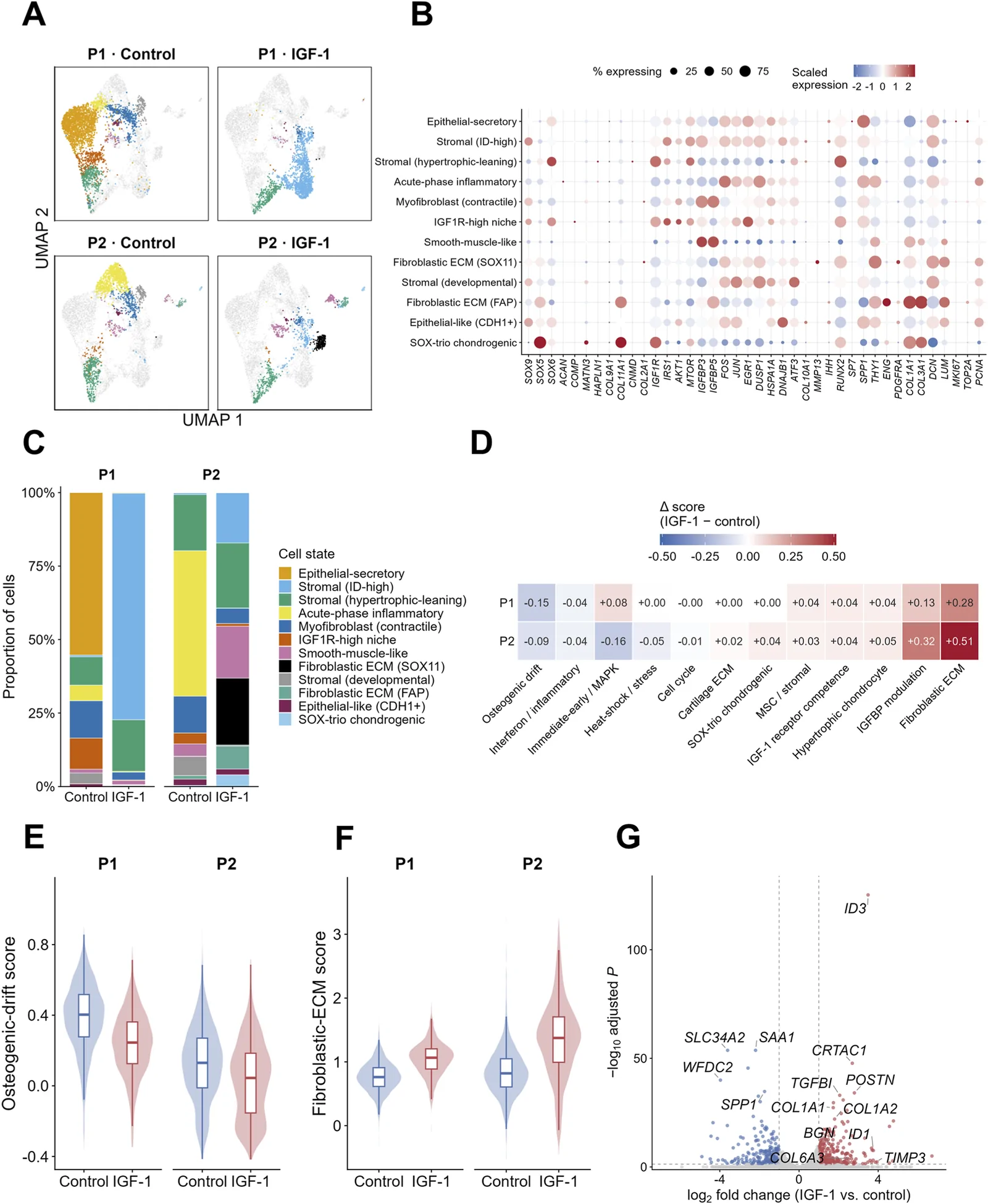
Single-cell RNA sequencing shows IGF1-associated changes in osteogenic and extracellular-matrix cell states. **(A)** UMAP embedding of chUDC single-cell transcriptomes for P1 and P2 under paired control and IGF1-treated conditions, colored by annotated cell state. **(B)** Dot plot of marker genes used to annotate the 12 chUDC states; dot size encodes the percentage of expressing cells and color the scaled mean expression. **(C)** Relative cell-state composition for P1 and P2 under control and IGF1 conditions. **(D)** Heatmap of module-score differences (IGF1 − control) across cell-state programs for P1 and P2. **(E)** Osteogenic module scores and **(F)** fibroblastic-ECM module scores in control versus IGF1-treated cells for P1 and P2. Cell-level module-score distributions are shown descriptively for each donor; no donor-level inferential testing was performed because only two donor-derived lines were available. **(G)** Volcano plot of differential expression (IGF1 vs. control); upregulated transcripts include *COL1A1*, *POSTN*, *CRTAC1*, *TGFBI*, *ID1* and *ID3*, and downregulated transcripts include *SPP1*, *SAA1*, *WFDC2* and *SLC34A2*.

UMAP visualization showed that *COL2A1*-mutant chUDCs occupied multiple transcriptionally distinct regions that were differentially represented across donors and treatment conditions (Figure 3A). Unsupervised clustering and marker-based annotation identified 12 chUDC states (Figure 3B), including SOX-trio-associated chondrogenic, fibroblastic extracellular-matrix, *IGF1R*-high, contractile/myofibroblast-like, inflammatory or epithelial-like, and osteogenic-associated states. Complete annotations, marker tables, and sample-level state composition summaries are provided as Supplementary Table S5.

IGF1 co-treatment altered the relative distribution of states in both patient-derived lines, although individual state changes differed between P1 and P2 (Figure 3C). Module-score analysis showed reduced osteogenic-associated scores and increased fibroblastic ECM and IGFBP-modulation scores after IGF1 exposure (Figures 3D–F). Extended module-score plots and donor-level module-score deltas are provided in Supplementary Figures S3A, B and Supplementary Table S6.

Differential expression analysis comparing IGF1-treated and control cells supported this state-level interpretation. IGF1 increased a set of ECM-associated and regulatory genes, including *COL1A1*, *COL1A2*, *POSTN*, *CRTAC1*, *TGFBI*, *ID1*, *ID3*, and *TIMP3*, while reducing *SPP1*, *SAA1*, *WFDC2*, and *SLC34A2* (Figure 3G). Selected transcripts within this response have documented roles in cartilage extracellular-matrix, matricellular, osteogenic/acute-phase, or epithelial programs (Steck et al., 2007; Mosher et al., 2015; Si et al., 2020; Sack, 2018; Bingle et al., 2006; Saito et al., 2015). The complete IGF1-response output is provided as Supplementary Table S7.

Because IGF1 markedly altered state composition, we asked whether the response was also cell-intrinsic. Per-state pseudobulk testing was possible in the three states retaining sufficient cells in all donor-by-treatment groups and identified 711, 566 and 9 differentially expressed genes in the myofibroblast-like, hypertrophic-leaning stromal and smooth-muscle-like states. *COL1A1*, *ID3*, *SERPINE1*, *SPP1*, *TGFBI* and *TNFSF10* responded consistently across all three (Supplementary Table S10; Supplementary Figure S5). The IGF1-associated shift therefore reflects both changed state proportions and transcriptional change within states.

Because single-cell analysis was limited to two patient-derived lines, these findings should be interpreted as proof-of-concept evidence for IGF1-responsive transcriptional programs rather than therapeutic rescue or clinical growth-factor-response prediction.

## 4 Discussion

In this proof-of-concept study, patient-derived *COL2A1*-mutant chUDCs showed transcriptional differences that were not evident by conventional differentiation staining.

This bulk signature was limited rather than broad and should not be interpreted as full growth-plate or skeletal-dysplasia recapitulation *in vitro*. Instead, it indicates that a non-invasively accessible, chondrogenically induced patient-derived system can retain selected transcriptional programs relevant to cartilage matrix biology and endochondral growth (Eyre, 2002; Mackie et al., 2008).

*COL2A1*-associated disease is primarily structural and matrix-related rather than primarily transcriptionally regulatory, and our data are most consistent with chUDCs reporting a downstream consequence of abnormal collagen expression rather than a genotype-specific transcriptional driver (Bateman et al., 2009). The robust elements of the signature support this reading: *DDR2* encodes a collagen receptor, whereas *ADAMTS5* encodes the principal aggrecanase, so the differential expression describes altered cell-matrix signaling and matrix turnover. We found no unfolded-protein or collagen-folding stress response at the transcript level, with no gene in either module reaching significance (Supplementary Table S9), suggesting that the measurable consequence in this two-dimensional system is matrix remodeling rather than intracellular stress. The model is therefore best understood as a system that detects matrix-remodeling consequences of abnormal collagen expression, not as a readout of variant-specific mechanism.

Single-cell RNA sequencing refined this phenotype by showing that chUDCs comprise discrete transcriptional states, consistent with heterogeneity reported in other MSC-based chondrogenic cultures (Chun et al., 2024), although single-cell studies of expanded chondrocytes have yielded contrasting results across cell sources and culture systems (Karlsen et al., 2021).

The IGF1 experiment extends this model toward endocrine growth biology (Racine and Serrat, 2020; Dixit et al., 2021). In the two patient-derived lines analyzed by single-cell RNA sequencing, IGF1 co-treatment was associated with the state and gene-level changes detailed above, supporting transcriptional modulation toward fibroblastic extracellular-matrix programs.

The three patient-derived lines were selected to sample a clinically meaningful spectrum of pathogenic *COL2A1* variants rather than a single genotype: one frameshift variant causing Stickler syndrome (P1) and two glycine-substitution variants associated with disproportionate short stature (P2 and P3). Triple-helical glycine substitutions and truncating variants represent mechanistically distinct classes of type II collagenopathy with variable skeletal and ocular phenotypes (Barat-Houari et al., 2016). Pooling mechanistically distinct variants tests the convergent, variant-independent component of the phenotype and is conservative rather than permissive, because averaging across distinct mechanisms dilutes rather than creates signal. That all three lines moved in the same direction for every signature gene, without overlap between patient and control ranges, indicates a convergent signature rather than a single-genotype effect.

Chondrogenic induction was performed as a two-dimensional monolayer rather than in a pellet, micromass, or organoid format. Although three-dimensional architecture and mechanical cues influence chondrogenic maturation and hypertrophy, a scalable and uniform two-dimensional system was better suited to the non-invasive, multi-donor single-cell comparison of rare private variants, reducing pellet-to-pellet variability and cell-number requirements. Three-dimensional and organoid systems remain an important complementary approach for assessing matrix architecture and zonal organization in future work (Lamande et al., 2023; Statham et al., 2022).

The study has limitations inherent to its proof-of-concept design. Bulk RNA sequencing included three patient-derived and three control lines, and single-cell RNA sequencing was limited to two patient-derived lines under paired control and IGF1-treated conditions. The cohort includes different *COL2A1* variants and phenotypes, which restricts genotype-specific conclusions, and controls were not age-matched to patients; because the control cultures used for these ancillary experiments differed from the patient cultures in their transient ROCK-inhibitor exposure, the between-group qPCR comparison and the qualitative type II collagen immunofluorescence are regarded as supportive rather than definitive validation. ChUDCs are non-invasive and experimentally tractable, but are not equivalent to native growth plate chondrocytes and do not reproduce growth-plate architecture, mechanical environment, endocrine context, or developmental zonation (Statham et al., 2022; Agirdil, 2020). The single-cell IGF1 experiment did not include IGF1-treated control lines; the paired within-donor design isolates the IGF1-associated shift within each patient line but cannot separate disease-specific from general IGF1 effects. The observed shift is equally compatible with a generic response of chondrogenically induced mesenchymal stromal-like cells to IGF1, and no claim of *COL2A1* specificity is made. IGF-axis regulators differed nominally between groups at baseline (*PAPPA2*, *IGFBP1*, *PAPPA* and *STC1*, each nominal p < 0.05 but not significant after correction), which motivated probing this axis but does not establish specificity. Single-cell profiling of IGF1-treated control-donor chUDCs is the priority next step. Direct type II collagen immunofluorescence and Alcian blue proteoglycan quantification provide initial protein- and matrix-level anchors, whereas protein-level validation of individual matrix-remodeling effectors such as *DDR2*, *DCN*, and *LOX* remains an important next step. Each donor was represented by a single independent culture and differentiation. Primary urine-derived material from affected children is limited, and the design therefore prioritized donor breadth over within-donor replication, because inter-individual variability dominates the variance in primary cultures. Reproducibility across independent cultures was therefore not assessed; the sensitivity analyses address donor-level rather than culture-level stability.

In conclusion, patient-derived *COL2A1*-mutant chUDCs show extracellular-matrix and ossification-related transcriptional differences that were not apparent from conventional staining and were modulated by IGF1 co-treatment. This study supports chUDCs as a non-invasive system for exploring selected cartilage- and growth-factor-responsive programs in *COL2A1*-associated growth disorders.

## Data Availability

The datasets presented in this study can be found in online repositories. The names of the repository/repositories and accession number(s) can be found in the article/Supplementary Material.
