# Supplementary material for: Donor-dependent heterogeneity and matrix-remodeling transcriptional programs in COL2A1-variant urine-derived cell cultures with IGF1-associated cell-state shifts
Source: Front Cell Dev Biol. 2026 Sep 1;14:1920598. doi: 10.3389/fcell.2026.1920598 (PMC13574590; doi:10.3389/fcell.2026.1920598)
Supplement: Supplementary file 1 [file DataSheet1.docx]

**Supplementary Material**

**Supplementary Data**

Supplementary material should be uploaded separately on submission. The supplementary tables listed below are provided as separate editable Excel workbooks. The supplementary figures and legends are included in this file.

**Supplementary Tables**

| **Item** | **File** | **Content** |
| --- | --- | --- |
| Supplementary Table 1 | Supplementary_Table_1_Donor_Metadata.xlsx | Donor/sample metadata, including group, sex, age at sampling, *COL2A1* genotype, phenotype, and sequencing arms. |
| Supplementary Table 2 | Supplementary_Table_2_Bulk_DESeq2_PatientVsControl.xlsx | Bulk RNA-seq DESeq2 results for patient-derived versus control chUDCs, including all genes, significant genes, directional subsets, and normalized counts. |
| Supplementary Table 3 | Supplementary_Table_3_Bulk_GSEA_PatientVsControl.xlsx | Gene Ontology Biological Process gene-set enrichment analysis results for patient-derived versus control chUDCs. |
| Supplementary Table 4 | Supplementary_Table_4_scRNA_QC_Integration.xlsx | Single-cell RNA-seq quality-control filtering, doublet summaries, library metrics, dimensionality-reduction metrics, donor mixing entropy, and per-sample metrics. |
| Supplementary Table 5 | Supplementary_Table_5_scRNA_CellStates_Markers_Composition.xlsx | Curated cell-state annotations, state composition, donor/condition/sample percentages, top markers, and full cluster marker table. |
| Supplementary Table 6 | Supplementary_Table_6_scRNA_ModuleScores.xlsx | Module gene sets and IGF1-responsive module-score summaries, including donor-level deltas, exploratory within-donor Wilcoxon tests, and within-state deltas. |
| Supplementary Table 7 | Supplementary_Table_7_scRNA_IGF1_Response.xlsx | IGF1-responsive state-composition deltas, exploratory within-donor Fisher tests, and pseudobulk differential-expression results for IGF1-treated versus control chUDCs. |
| Supplementary Table 8 | Supplementary_Table_8_Signature_Robustness.xlsx | Robustness of the bulk patient-versus-control signature: leave-one-donor-out DESeq2 models, effect-size and rank stability, per-donor expression values with control/patient ranges, per-sample outlier diagnostics (Cook's distance, sample correlations, PCA coordinates), and top-100 DEG overlap per run. |
| Supplementary Table 9 | Supplementary_Table_9_Age_and_ERstress.xlsx | Age sensitivity analysis (quantification of the age-condition confounding; signature genes before and after adjustment for age as a continuous variable and as a pediatric/adult factor) and unfolded-protein-response / collagen-folding module analysis with per-gene results and per-sample module scores. |
| Supplementary Table 10 | Supplementary_Table_10_Pseudobulk_PerState_IGF1.xlsx | Per-cell-state pseudobulk differential expression for IGF1-treated versus untreated chUDCs (edgeR quasi-likelihood, donor-paired design), with cell numbers per donor, state and treatment, testability criteria, and per-state result tables. |
| Supplementary Table 11 | Supplementary_Table_11_Deconvolution_Sensitivity.xlsx | Compositional sensitivity analysis: cell-state proportions estimated in each bulk sample by non-negative least squares against a single-cell derived signature matrix, between-group tests of the estimated proportions, and signature genes before and after composition adjustment. |

**Supplementary Notes**

**Supplementary Note 1. Interpretation of epithelial-secretory, acute-phase inflammatory, and epithelial-like states.** Three annotated states outside the main chondrogenic/stromal continuum require cautious interpretation. The epithelial-secretory state, defined by *SLPI*, *WFDC2*, *SCTR*, *SLC34A2*, and *NAPSA*, was strongly enriched in the untreated P1 culture, accounting for 55.3% of cells, but was rare in the other samples. The acute-phase inflammatory state, defined by *FGB*, *LCN2*, *SAA1*, *SAA2*, *C1R*, and *CXCL14*, was strongly enriched in untreated P2, accounting for 49.5% of cells, and was less abundant in the other samples. A separate *CDH1*-positive epithelial-like state remained minor across samples, accounting for approximately 0.3–2.1% of cells. The coherent epithelial-secretory signature may reflect retained urothelial or renal-tubular populations originating from the urinary starting material or culture-associated epithelial state programs, whereas the acute-phase signature may reflect donor- or culture-specific inflammatory or stress activation. Contributions from ambient RNA or other technical effects cannot be excluded, but the present data do not allow these possibilities to be distinguished from genuine culture subpopulations. We therefore treat these states as donor- and culture-system-associated observations rather than validated differentiation trajectories or disease-specific populations. Complete marker sets and sample-level state compositions are provided in Supplementary Table 5.

**Supplementary Figures**

**Supplementary Figure 1. Single-cell RNA-seq quality control and doublet filtering.** (A) Distributions of UMIs per cell, genes per cell, and mitochondrial read fraction before raw-cell filtering and after quality-control filtering for P1, P1 IGF1, P2, and P2 IGF1. (B) Percentage and number of cells retained after quality-control filtering. (C) Percentage and number of predicted doublets per sample after scDblFinder classification. (D) UMAP projection of the merged dataset colored by scDblFinder doublet score, illustrating the doublet-score landscape before integrated singlet analysis.

**Supplementary Figure 2. Single-cell RNA-seq integration and dimensionality-reduction diagnostics.** (A) UMAP projection before integration colored by donor, showing donor-driven separation. (B) UMAP projection after integration colored by donor, showing improved overlap between P1 and P2. (C) Mean normalized donor mixing entropy per cluster before integration using PCA, after full integration, and after singlet filtering. (D) Principal component standard deviations used for dimensionality selection; the first 20 PCs were used for downstream analysis. (E, F) PCA projection after filtering and integration colored by donor (E) and condition (F), showing residual biological structure without complete donor- or treatment-driven segregation.

**Supplementary Figure 3. Extended IGF1-responsive module-score analysis and mapping of bulk RNA-seq genes to single-cell states.** (A) Violin/box plots of module-score distributions across control and IGF1-treated P1 and P2 chUDCs for all scored programs. P values indicate exploratory within-donor Wilcoxon rank-sum tests and are interpreted as cell-level distributional summaries rather than independent donor-level evidence. (B) IGFBP-modulation module scores in control and IGF1-treated P1 and P2 chUDCs. (C) Mapping of selected bulk RNA-seq patient-associated genes to annotated single-cell states; the x-axis shows bulk log2 fold change for patient-derived versus control chUDCs, and labels indicate major single-cell states in which the corresponding transcript was identified as a marker. *DDR2* is shown as bulk-regulated but was not recovered as a single-cell state marker.

Supplementary Figure 4. Robustness of the bulk patient-versus-control signature. (A) Log2 fold change of the seven signature genes in the full model and in each of the six leave-one-donor-out models (column label indicates the donor removed); asterisks mark adjusted p < 0.05. Direction is retained in 6/6 models for every gene, whereas significance is lost in individual models for the lower-abundance effects, consistent with reduced power at n = 2 per group. (B) Variance-stabilised expression of each signature gene in every donor. All three patient lines deviate from the control mean in the expected direction for all seven genes, and patient and control ranges do not overlap. (C) Adjusted p values for the signature genes in the primary model, after adjustment for age, and after adjustment for estimated cell-state composition; the dashed line marks p = 0.05. Bars of zero height indicate an adjusted p value of approximately 1, reflecting the loss of residual degrees of freedom in the age-adjusted model, in which age and disease status are strongly collinear. Underlying values are given in Supplementary Tables 8, 9 and 11.

Supplementary Figure 5. Per-cell-state IGF1 response in patient-derived chUDCs. (A) Number of cells recovered for each cell state, donor and treatment condition; the dashed line marks the 20-cell threshold required in all four donor-by-treatment groups for a state to be testable. IGF1 substantially redistributes cells across states, so only three of twelve states meet this criterion. (B) Number of differentially expressed genes (FDR < 0.05) between IGF1-treated and untreated cells within each testable state, from pseudobulk profiles aggregated per donor, state and treatment and tested with a donor-paired design. A substantial cell-intrinsic response persists once composition is held constant, indicating that the IGF1-associated shift is not explained by redistribution between states alone. Because only two donors were profiled, these results are descriptive. Full results are given in Supplementary Table 10.
